# InTAD: chromosome conformation guided analysis of enhancer target genes

**DOI:** 10.1186/s12859-019-2655-2

**Published:** 2019-01-31

**Authors:** Konstantin Okonechnikov, Serap Erkek, Jan O. Korbel, Stefan M. Pfister, Lukas Chavez

**Affiliations:** 1 Hopp-Children’s Cancer Center at the NCT Heidelberg (KiTZ), Heidelberg, Germany; 2 Pediatric Neurooncology, German Cancer Research Center, Heidelberg, Germany; 3 Genome Biology Unit, European Molecular Biology Laboratory (EMBL), Heidelberg, Germany; 4 Izmir Biomedicine and Genome Center, Izmir, Turkey; 5 Department of Pediatric Hematology and Oncology, Heidelberg University Hospital, Heidelberg, Germany; 6 Department of Medicine, Division of Medical Genetics, University of California San Diego (UCSD), San Diego, USA

**Keywords:** Epigenomics, Transcriptomics, Topologically associated domains, Enhancers

## Abstract

**Background:**

High-throughput technologies for analyzing chromosome conformation at a genome scale have revealed that chromatin is organized in topologically associated domains (TADs). While TADs are relatively stable across cell types, intra-TAD activities are cell type specific. Epigenetic profiling of different tissues and cell-types has identified a large number of non-coding epigenetic regulatory elements (‘enhancers’) that can be located far away from coding genes. Linear proximity is a commonly chosen criterion for associating enhancers with their potential target genes. While enhancers frequently regulate the closest gene, unambiguous identification of enhancer regulated genes remains to be a challenge in the absence of sample matched chromosome conformation data.

**Results:**

To associate enhancers with their target genes, we have previously developed and applied a method that tests for significant correlations between enhancer and gene expressions across a cohort of samples. To limit the number of tests, we constrain this analysis to gene-enhancer pairs embedded in the same TAD, where information on TAD boundaries is borrowed from publicly available chromosome conformation capturing (‘Hi-C’) data. We have now implemented this method as an R Bioconductor package ‘InTAD’ and verified the software package by reanalyzing available enhancer and gene expression data derived from ependymoma brain tumors.

**Conclusion:**

The open-source package InTAD is an easy-to-use software tool for identifying proximal and distal enhancer target genes by leveraging information on correlated expression of enhancers and genes that are located in the same TAD. InTAD can be applied to any heterogeneous cohort of samples analyzed by a combination of gene expression and epigenetic profiling techniques and integrates either public or custom information of TAD boundaries.

**Electronic supplementary material:**

The online version of this article (10.1186/s12859-019-2655-2) contains supplementary material, which is available to authorized users.

## Background

New technologies for analyzing the three-dimensional chromosome organization in a genome-wide manner have revealed mechanisms by which chromosome communication is established [1]. By using different types of high-throughput techniques, such as ChIP-sequencing sensitive for different types of histone modifications, whole genome bisulfite sequencing, ATAC-sequencing, and DNase-Seq, many studies have discovered a large number of enhancers involved in gene regulation. Importantly, the analysis of active chromatin can uncover potential targets relevant for precision treatment of cancer [2]. To associate enhancers with their target genes in the absence of sample-matched chromosome conformation data, several computational methods have been developed.

A widely used approach to associate enhancers with their target genes is to consider the closest genes along the linear DNA. For example, the R package ELMER uses 450 K DNA methylation array data to first define enhancers based on hypo-methylated CpGs and then predicts enhancer target genes by computing the correlation between DNA methylation and gene expression restricting the analysis to the 10 closest genes up- and downstream of the enhancer [3]. Another example is TENET, an analytical approach that associates genome-wide expression changes of transcription factors with gain or loss in enhancer activities by correlating DNA methylation levels at enhancers with the gene expression of transcription factors [4]. However, both tools require DNA methylation array data as input and restrict the correlation to the ‘closest genes’ or to transcription factors that regulate enhancers.

The 11-zinc finger DNA-binding protein CCCTC-binding factor (CTCF) plays an important role in chromatin organization [5]. To improve the identification of gene-enhancer interactions, information on CTCF binding sites can be leveraged. The PreSTIGE method employs this strategy by accessing CTCF ChIP-seq data derived from 13 cell types [6]. Here, CTCF binding sites are considered as insulators separating enhancers from their target genes. This method is currently available as an online application, however, its functionality is limited to the available reference data only and each sample is analyzed independently.

A fundamental concept of chromatin organization is topologically associated domains (TADs). TADs are segments of the genome characterized by frequent chromosome interactions within themselves and they are insulated from adjacent TADs [7]. It has been shown that mutations disturbing the integrity of TADs can lead to the activation of proto-oncogenes causing tumor development [8, 9].

**Fig. 1:**
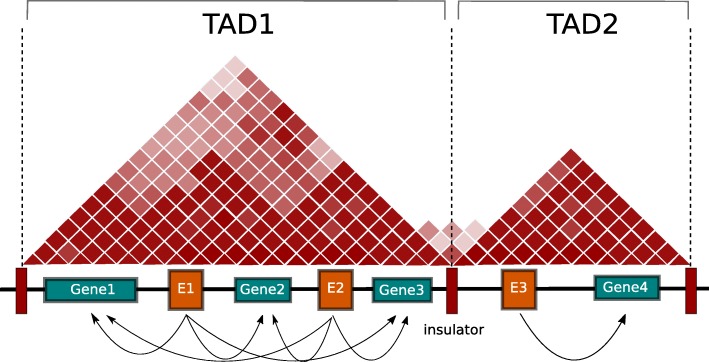
Chromatin is organized in topologically associated domains (TADs). The InTAD software package tests for significant correlations between genes and enhancers restricted by TAD boundaries

We have developed an R package, InTAD, that tests for significant correlations between genes and enhancers co-located in the same TAD (Fig. 1). Previously we employed this strategy to identify and validate enhancer-associated genes in different pediatric brain tumor types including medulloblastoma (*n* = 25 samples) [10], atypical teratoid/rhabdoid tumors (*n* = 11 samples) [11] and ependymoma (*n* = 24 samples) [12]. Importantly, InTAD is not restricted to specific data types and can detect enhancer-gene correlations in any cohort of samples analyzed by genome-wide gene expression and epigenetic profiling. While this approach cannot entirely compensate for the lack of condition-specific chromosome conformation data, it can predict proximal and distal enhancer target genes without limiting the analysis to the ‘closest gene’. The package is open-source and available at Bioconductor.

### Implementation

**Fig. 2:**
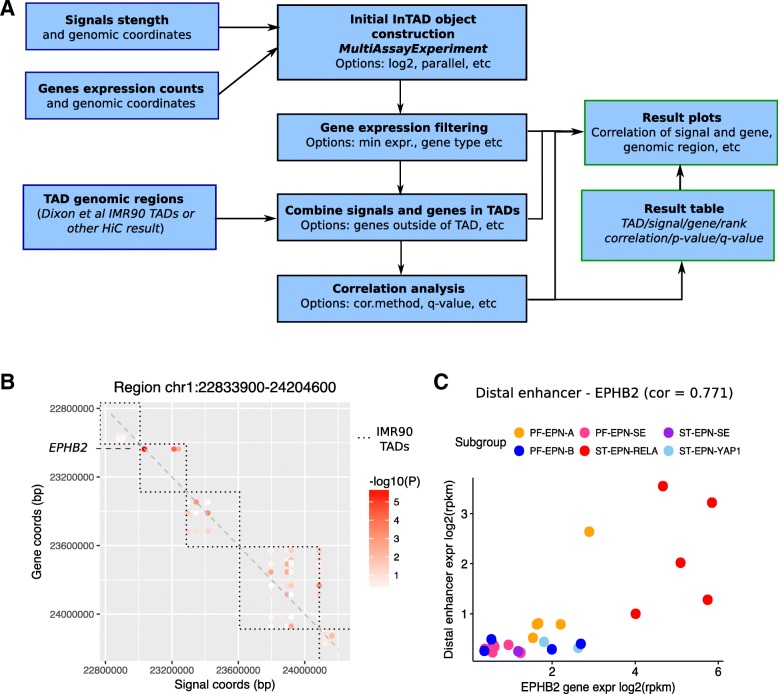
**a** Structure of the InTAD package. **b** Simulated Hi-C map based on correlations between enhancers (x-axis) and genes (y-axis). TAD boundaries are indicated as dashed boxes. Marked is EPHB2, a validated ependymoma oncogene that correlates significantly with proximal and distal enhancers. **c** The correlation plot reveals co-activation of EPHB2 and a distal enhancer element located 200 kbp away from the transcription start site. Both, EPHB2 and the distal enhancer element, are specifically expressed in ependymomas of the molecular subgroup ST-EPN-RELA

The structure of the InTAD package is outlined in Fig. 2a. InTAD requires three input data sets including a data matrix of epigenetic signals (e.g. normalized RPKM values at predefined enhancers derived from ChIP-seq data) and a gene expression matrix (e.g. normalized RPKM values from RNA-seq data). To identify enhancers and genes co-located in the same TAD, each data matrix has to contain the genomic coordinates of the enhancers or genes, respectively. The input data can be provided either as standard R objects, such as data frame, or as paths to the text files in common formats for count tables and genomic annotations. The function that generates the central data object performs inconsistency checks of the input data and provides various options, such as multi-core data processing to increase the performance. As indicated in Fig. 2a, the analysis starts by initialization of a MultiAssayExperiment R object [13].

Moreover, InTAD requires a predefined set of TAD regions as input. Since approximately 60–80% of TADs remain stable across cell types [14], the package comes with a set of TADs derived from IMR90 human fibroblast cell lines [7], which we have accessed in previous studies [10–12]. However, to take into account cell-type specific TAD boundaries, other HiC data can also be integrated by providing the resulting TAD regions as input in BED format.

Various parameters allow to control further steps of the analysis workflow. Genes can optionally be filtered based on the analysis of their expression distribution or by selecting specific types of RNA. Further, enhancers and genes are combined when their genomic coordinates are embedded in the same TAD. Since the boundaries of TADs have shown to be sensitive to the analytical method applied and may vary across cell types, genes that do not fall into a TAD are assigned to the nearest TAD by default. Subsequently, correlations between all enhancer-gene pairs within the same TAD are computed by selecting one of the supported methods: Pearson, Kendal or Spearman correlation. In addition, adjusted *p*-values can be calculated to control the false discovery rate using the R/Bioconductor package *qvalue* [15]. The final result table includes detailed information about the computed correlation values, adjusted p-values, and Euclidian distances as an additional measure that allows to identify potential correlations that suffer from scale invariance.

The results can be visualized by simulated Hi-C maps highlighting significant correlations at selected genomic loci (Fig. 2b). Additionally, correlations between a selected gene and enhancer pair can be visualized with custom colors by providing annotations that reflect groups of samples (Fig. 2c).

## Results

### Integration of TAD boundaries improves the identification of enhancer target genes

We have accessed H3K27ac ChIP-seq and RNA-seq data from our previous enhancer mapping study in ependymoma tumors [13] and verified our previous results by repeating the analysis using our new InTAD software package.

To estimate the dependency between the fraction of enhancer associated genes that can be identified by a given number of samples, we have performed a saturation analysis using our cohort of *n* = 24 ependymoma tumors. In each iteration, ranging from *n* = 10 to *n* = 23, we randomly sampled an according number of tumor samples, identified enhancer associated genes (EAG) using our InTAD software, and compared the number of retrieved EAGs to the number of EAGs obtained when using the entire cohort of n = 24 ependymoma tumors. As a result, we observe a saturation of identified EAGs starting at approximately 16 samples and more than ~ 95% of all EAGs were retained using at least 19 samples (Additional file 1: Figure S1A).

**Fig. 3:**
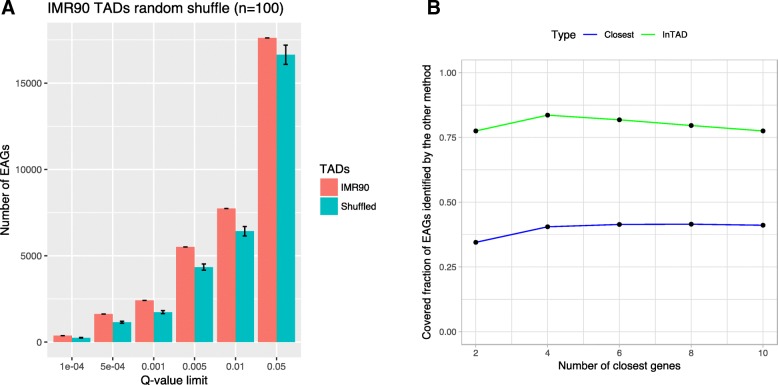
**a** Comparison of the number of enhancer associated genes obtained when considering IMR90 TADs compared to randomly generated TADs. The number of significantly correlated enhancer associated genes (EAGs) is constantly higher throughout the tested range of adjusted *p*-values when accessing the original TADs compared to random TADs. Error bars indicate standard deviation in the results of the 100 random sets of TADs. **b** EAGs annotated by both, InTAD and the “closest gene” approaches, are compared to each other across a varying range of the closest genes (2–10, x-axis). The mutual agreement of both approaches is shown on the y-axis as a covered fraction of detected enhancer associated genes from the results of the other approach

To further test the importance of TADs for the detection of enhancer-gene interactions, we repeated the same analysis using randomly generated TADs. The random TADs were designed by considering the genomic locations, lengths, and gaps between TADs obtained from IMR90 cells [7]. To create random TADs, we have randomized the order of these regions. This was repeated 100 times by changing the random seed from 1 to 100. In each iteration, the same correlation analysis of enhancers and genes in ependymoma tumors was performed as described above with the only difference of using the random sets of TADs instead. By applying adjusted *p*-value thresholds between 0.0001 to 0.1, we compared the number of significant correlations obtained when considering the original set of TADs versus the number of significant correlations obtained when considering the 100 random sets of TADs (Fig. 3a). As a result we consistently observe a higher number of significant enhancer-gene correlations when accessing the original TADs compared to random TADs across the entire range of tested significance thresholds. We further increased the number of permutations of TADs to 500 and plotted the resulting distributions of the number of significantly correlated enhancer target genes for six different q-value thresholds (Additional file 1: Figure S1B). In all but one cases, the number of EAGs identified when considering the original TADs is significantly (*p*-value <1e-10) larger than the number of EAGs identified using permutated TADs. These results provide further evidence for the importance of integrating experimentally derived TADs and justify our choice of an adjusted p-value threshold of 0.01 applied in our original analysis.

We were also interested in comparing the results of our enhancer-gene correlation method with results obtained when linking enhancers with the closest genes. Therefore, we have annotated the epenydmoma enhancers with the 2 to 10 closest genes located upstream and downstream of the enhancers. By considering an adjusted p-value threshold of 0.01 for our original InTAD correlation analysis, we compared enhancer associated genes detected by both methods (Fig. 3b). As a result, we observe that more than 50% of potential enhancer target genes are missed by the closest gene annotation, even though they are located in the same TAD and their gene expression is significantly correlated with the expression of enhancer elements. Notably, up to 75% of enhancer associated genes annotated by the closest gene approach are also identified by our correlation strategy. The majority (> 99%) of enhancer target genes that are only annotated by the closest gene approach are not located in the same TAD as the enhancer, rendering them as likely false positives.

### The inclusion of genes outside TADs increases the sensitivity in detecting enhancer target genes

**Fig. 4:**
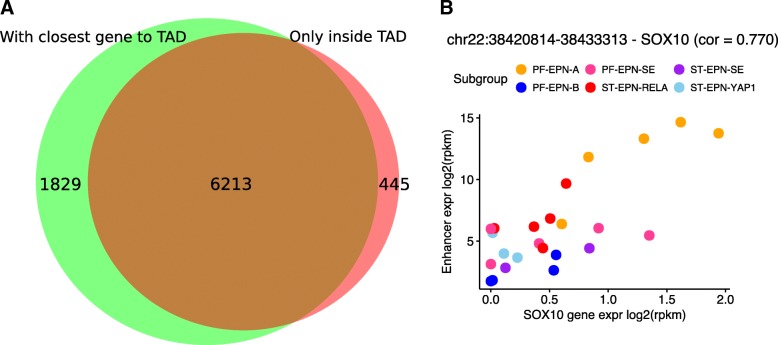
**a** Venn-diagram showing the number and overlap of enhancer associated genes identified in our original study compared to the re-analysis of the same data using InTAD by also considering genes located outside of TADs. **b** The transcription factor SOX10 is specifically active in ependymoma tumors of the subtype PF-EPN-A and significantly correlated with an enhancer element located 40,275 bp upstream of the SOX10 transcription start site. SOX10 was missed previously, because it is located outside of TADs obtained from IMR90 cells and was therefore not considered during the enhancer-gene correlation analysis

We have observed for different HiC data sets that several genomic regions are void of annotated TADs. Such regions can result from cell-type specific chromatin organization that renders some regions as inactive, or from artefacts introduced by sample preparation and HiC data analysis. To avoid neglecting genes located in regions outside of annotated TADs, especially when no sample or cell-type matched chromosome conformation data is available, we included the option to associate genes with their nearest TAD prior to the correlation analysis. By enabling this option, we re-analyzed the ependymoma data and compared the results to our original analysis [12]. As a result, ~ 93% of previously discovered enhancer target genes were confirmed using the same adjusted *p*-value of 0.01 (Fig. 4a). In addition, we detected 1829 potential new enhancer associated genes. These newly discovered genes were previously neglected, because they are located outside of the boundaries derived from IMR90 cells.

Among others, this novel approach revealed a strong enhancer element potentially regulating the transcription factor SOX10. SOX10 functions in neural crest and oligodendrocyte development and has previously been described controversially as a negative marker for the diagnosis of ependymoma tumors [16, 17]. Based on our re-analysis of the available gene expression and enhancer data across six intracranial ependymoma subgroups, we find that SOX10 is specifically expressed in the subgroup PF-EPN-A (Fig. 4b), likely regulated by a subgroup-specific enhancer element located ~ 40 Kbp upstream of the gene. These results indicate a tumor-specific chromosome conformation that potentially allows interactions between the PF-EPN-A specific enhancer element and the SOX10 gene. This example demonstrates the importance of the new functionality to allow the usage of empty regions between TADs, especially when accessing reference chromosome conformation data obtained from unrelated cell types.

### TADs derived from related cell-types improve the identification of EAGs

The discovery of TADs revealed global levels of stability of chromatin organization across cell types. However, recent studies show that up to 40% of TADs can differ between different tissues and organs [14]. Moreover, it has been shown that different computational methods for the analysis of TADs largely result in different numbers and lengths of TADs for the same data set [18, 19]. To further investigate the impact of the chosen reference chromosome conformation data, we repeated our analysis by using TADs obtained from cerebellum astrocytes provided by the ENCODE project [20]. We selected this cell type since it is expected to be more similar to brain tumors in comparison to the previously accessed IMR90 TADs. The total number of TADs and their mean length appeared to be largely similar between IMR90 and cerebellum astrocytes (Additional file 2: Figure S2A). The majority of EAGs (~ 75%) can be identified by considering any of the two different sets of TADs, however, by considering TADs obtained from cerebellum astrocytes, we identify noticeably more EAGs compared to TADs derived from IMR90 cells (7746 vs 6658, Additional file 2: Figure S2B). Moreover, by considering TADs from cerebellum astrocytes, we can identify additional known ependymoma marker genes as EAGs, such as for example SOX10, due to their co-location with enhancer elements in the same TAD. Importantly, correlations are in average higher between genes and enhancers co-located in TADs that are common in IMR90 and cerebellum astrocytes (Additional file 2: Figure S2C). Similarly, correlations are generally higher in TADs specific to cerebellum astrocytes in comparison to TADs specific to IMR90 cells, providing additional evidence for the relevance of choosing HiC data derived from related cell-types.

## Conclusions

In this study we present a novel R/Bioconductor package InTAD that allows to identify enhancer associated genes within and across TADs using epigenetic and transcriptomic data. In comparison to other existing tools, InTAD supports different input data types and overcomes the limits of the “closest gene” strategy by integrating information on TADs obtained from public or custom chromosome conformation analysis experiments. We have employed InTAD for the re-analysis of H3K27ac ChIP-seq and RNA-seq data obtained from 24 ependymoma brain tumors. Additionally, by performing simulation tests we confirmed the benefit of the TADs usage to identify enhancer associated genes based on the comparison to the application of random TADs. It’s important to note that the choice of a specific set of TADs will have an impact on the resulting number of enhancer target genes. If cell-type matched HiC data is unavailable, we recommend to use other publicly available TADs and to adjust the InTAD parameters to allow for the inclusion of genes outside TADs in order to increase the sensitivity. Moreover, there exist different analysis strategies and methods for calling TADs and commonalities and differences of these tools are still under debate in the field [18, 19]. The package also includes other options to control the sensitivity of the workflow such as filtering for lowly expressed genes, calculation of the Euclidian distance, and computation of adjusted *p*-values. In addition, InTAD allows to generate plots that show predicted chromosome conformation based on enhancer-gene correlations. We expect that InTAD will have a positive impact on future enhancer profiling studies focused on the identification and prioritization of oncogenes or important regulators of cell-type identity in health and disease.

### Availability and requirements

Project name: InTAD.

Project home page: https://github.com/kokonech/InTAD

Operating system(s): platform independent.

Programming language: R.

Other requirements: R 3.5.0 or higher, Bioconductor 3.7 or higher.

License: GNU GPL v2.

Any restrictions to use by non-academics: none.

## Additional files

Additional file 1: **Figure S1.** A) The proportion of recovered enhancer associated genes (EAG) as a function of random subsets of ependymoma tumor samples (correlation *p*-value 0.05). The random selection of subsamples was repeated 10 times in each iteration (*n* = 10 to *n* = 23) to derive the indicated mean and standard deviations. B) Distribution of EAGs obtained when considering random TADs repeated 500 times using adjusted p-value limits from 0.0001 to 0.05. Green vertical lines reflect the number of EAGs detected when considering experimentally derived TADs from IMR90 cells. In all cases the permutation test p-value is smaller than 1e-10, except for the correlation analysis using an adjusted p-value limit of 0.05 where the permutation test p-value equals 0.078. (PDF 159 kb)

Additional file 2: **Figure S2.** A) The variance between the sizes of TADs derived from IMR90 and cerebellum astrocytes. B) Venn-diagram showing the number and overlap of enhancer associated genes identified in ependymoma tumors using TADs derived from IMR90 or cerebellum astrocytes, respectively. C) Boxplot summarizing the enhancer-gene correlation values obtained when considering TADs common between IMR90 and cerebellum astrocytes or TADs specific to cerebellum astrocytes or IMR90, respectively. (PDF 88 kb)

## Abbreviations

EAG: Enhancer associated gene
EPN: Ependymoma brain tumor
RPKM: Reads per Per Kilobase of transcript, per Million mapped reads
TAD: Topologically associated domain
