# Supplementary figures and images for: InTAD: chromosome conformation guided analysis of enhancer target genes
Source: BMC Bioinformatics. 2019 Jan 31;20:60. doi: 10.1186/s12859-019-2655-2 (PMC6357397; doi:10.1186/s12859-019-2655-2)

**A**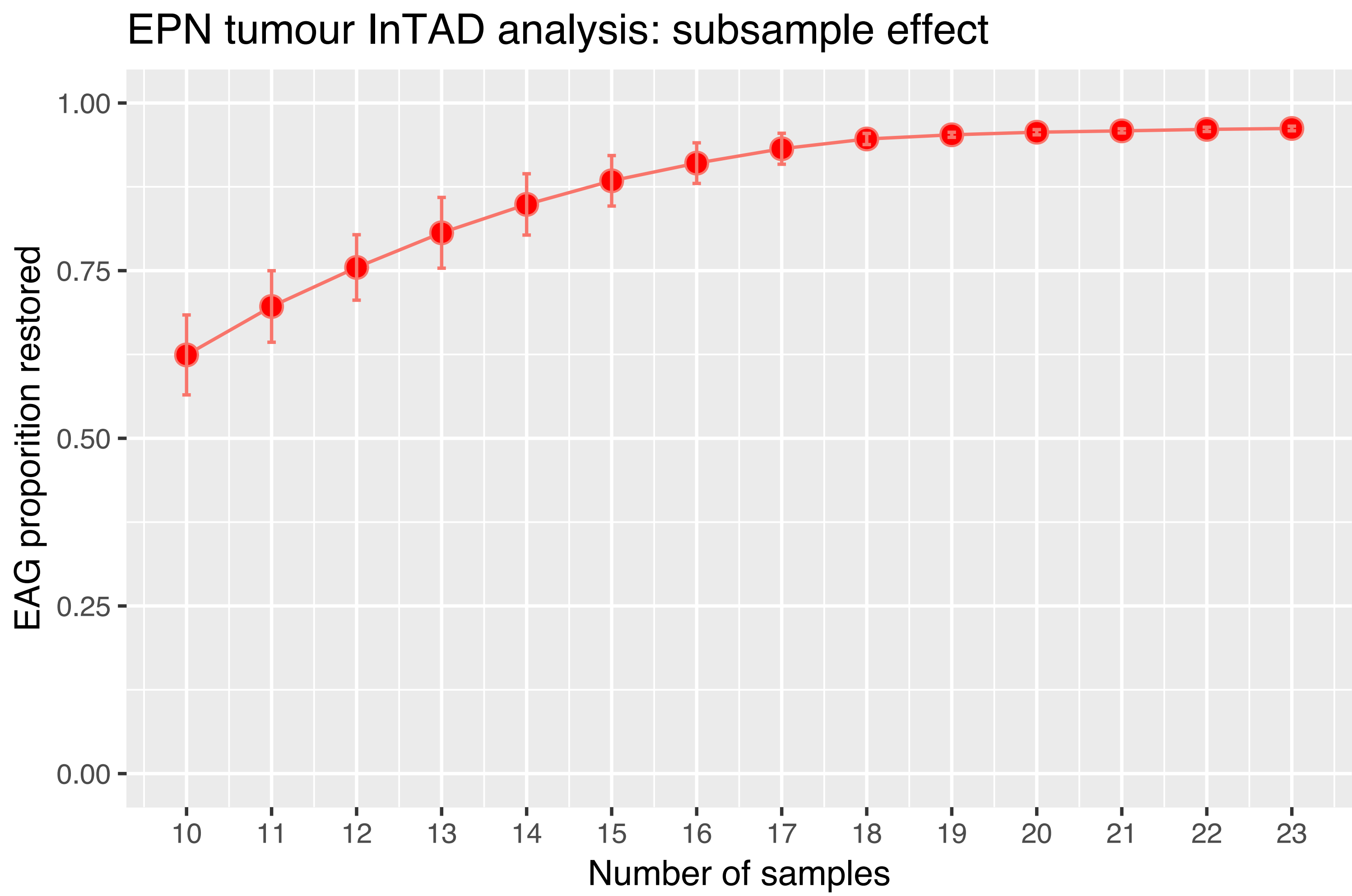**B**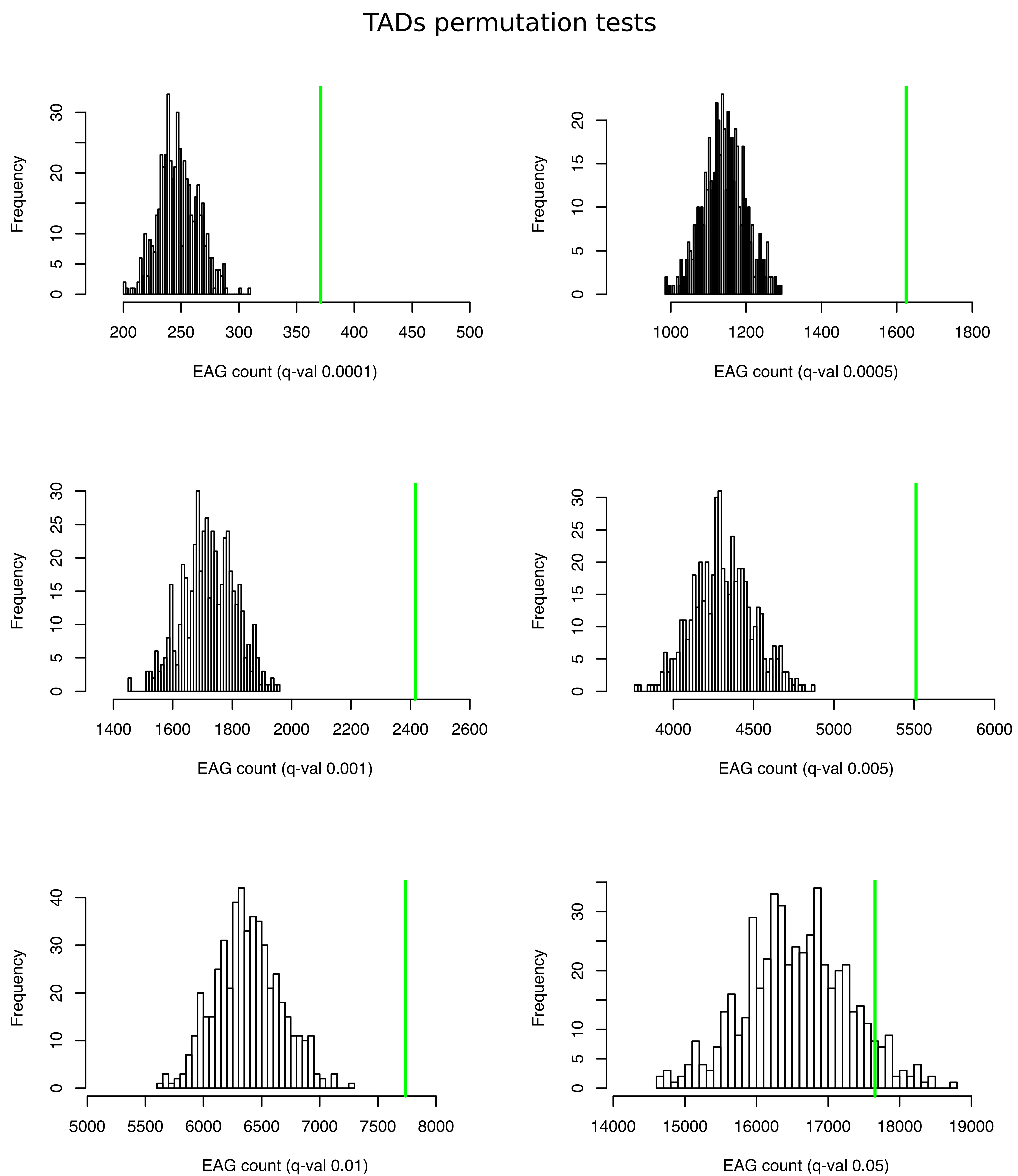

Supplement: Supplementary file 1 — Figure S1. A) The proportion of recovered enhancer associated genes (EAG) as a function of random subsets of ependymoma tumor samples (correlation p-value 0.05). The random selection of subsamples was repeated 10 times in each iteration (n = 10 to n = 23) to derive the indicated mean and standard deviations. B) Distribution of EAGs obtained when considering random TADs repeated 500 times using adjusted p-value limits from 0.0001 to 0.05. Green vertical lines reflect the number of EAGs detected when considering experimentally derived TADs from IMR90 cells. In all cases the permutation test p-value is smaller than 1e-10, except for the correlation analysis using an adjusted p-value limit of 0.05 where the permutation test p-value equals 0.078. (PDF 159 kb) [file 12859_2019_2655_MOESM1_ESM.pdf]

**A**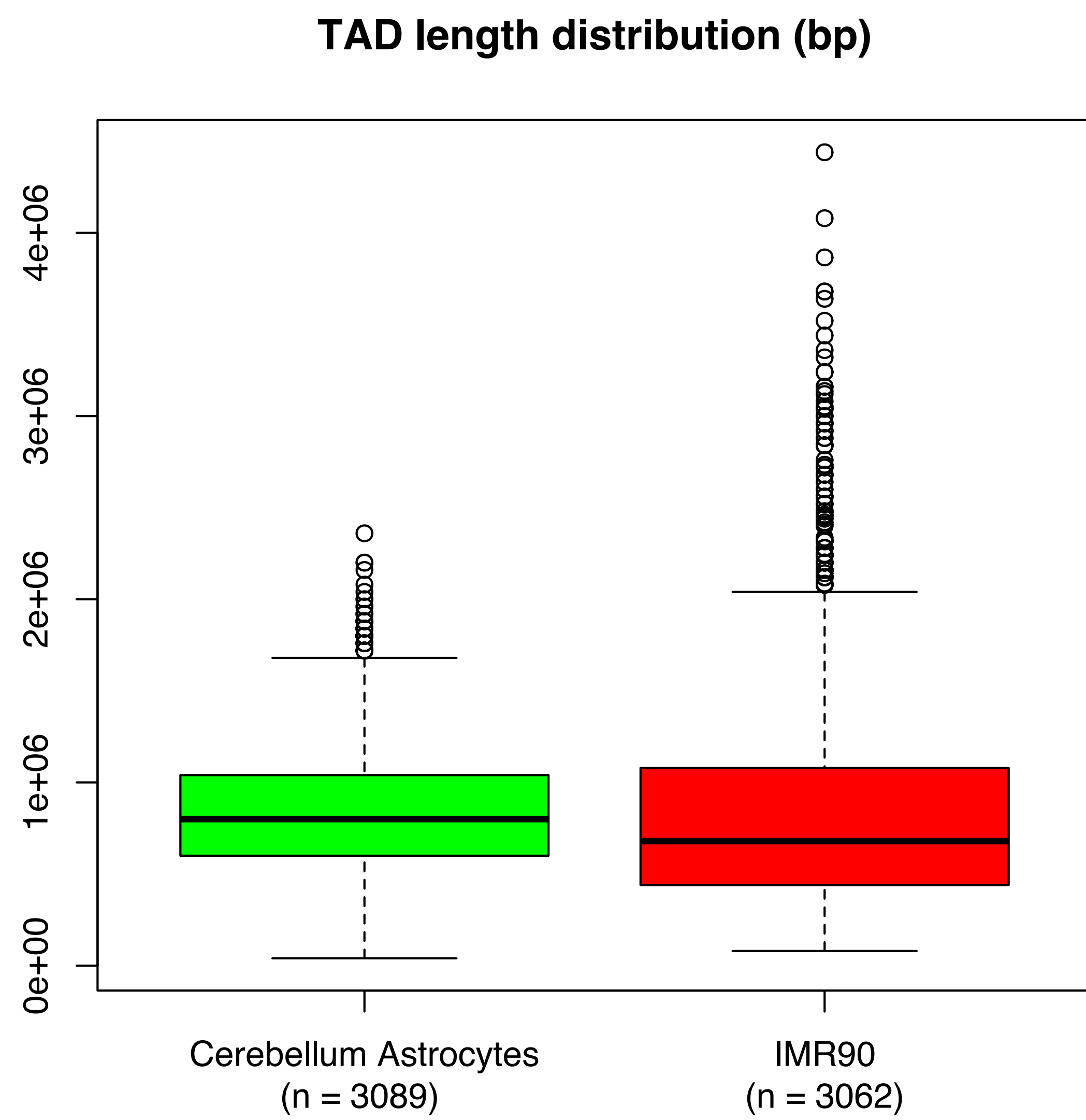**B**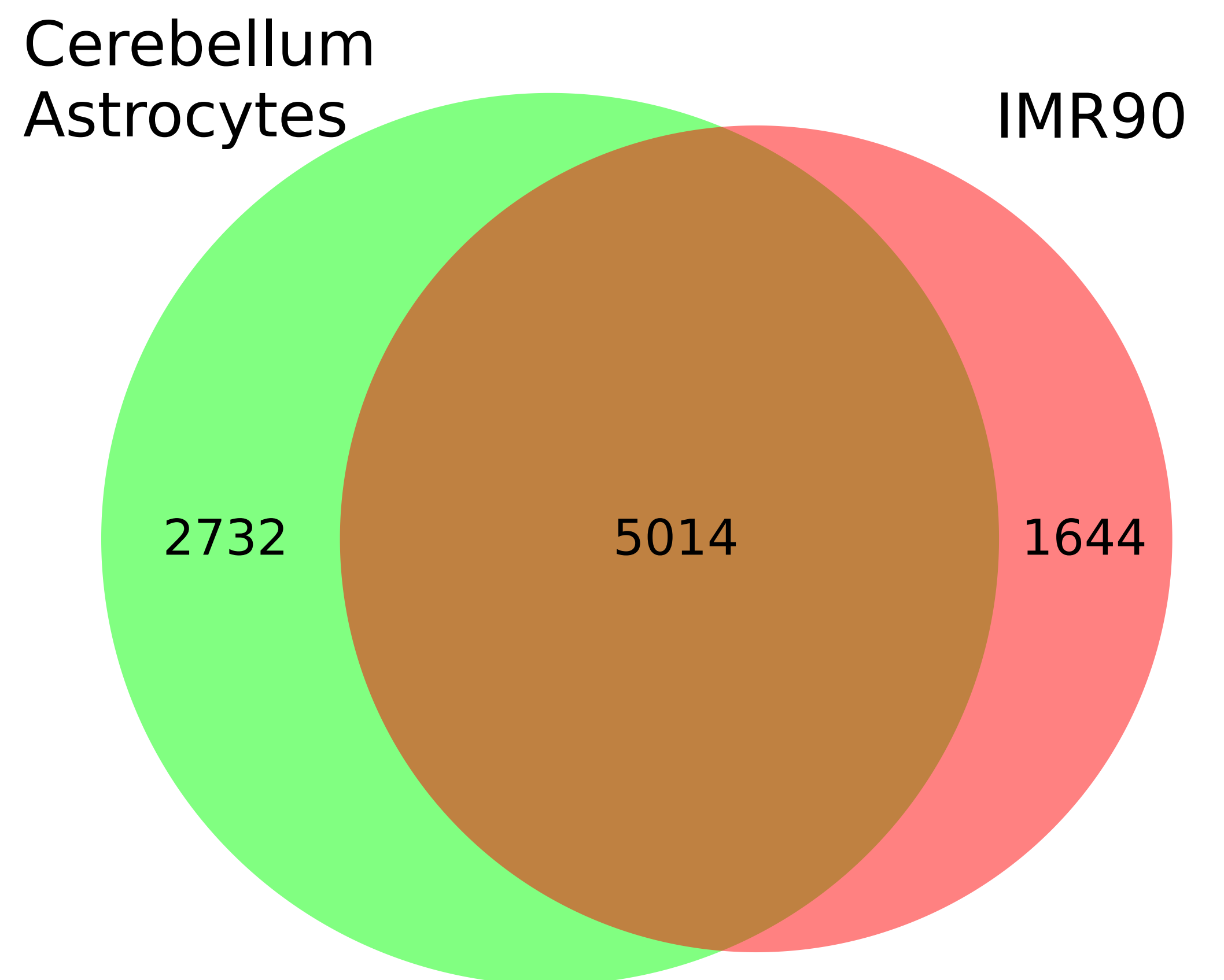**C**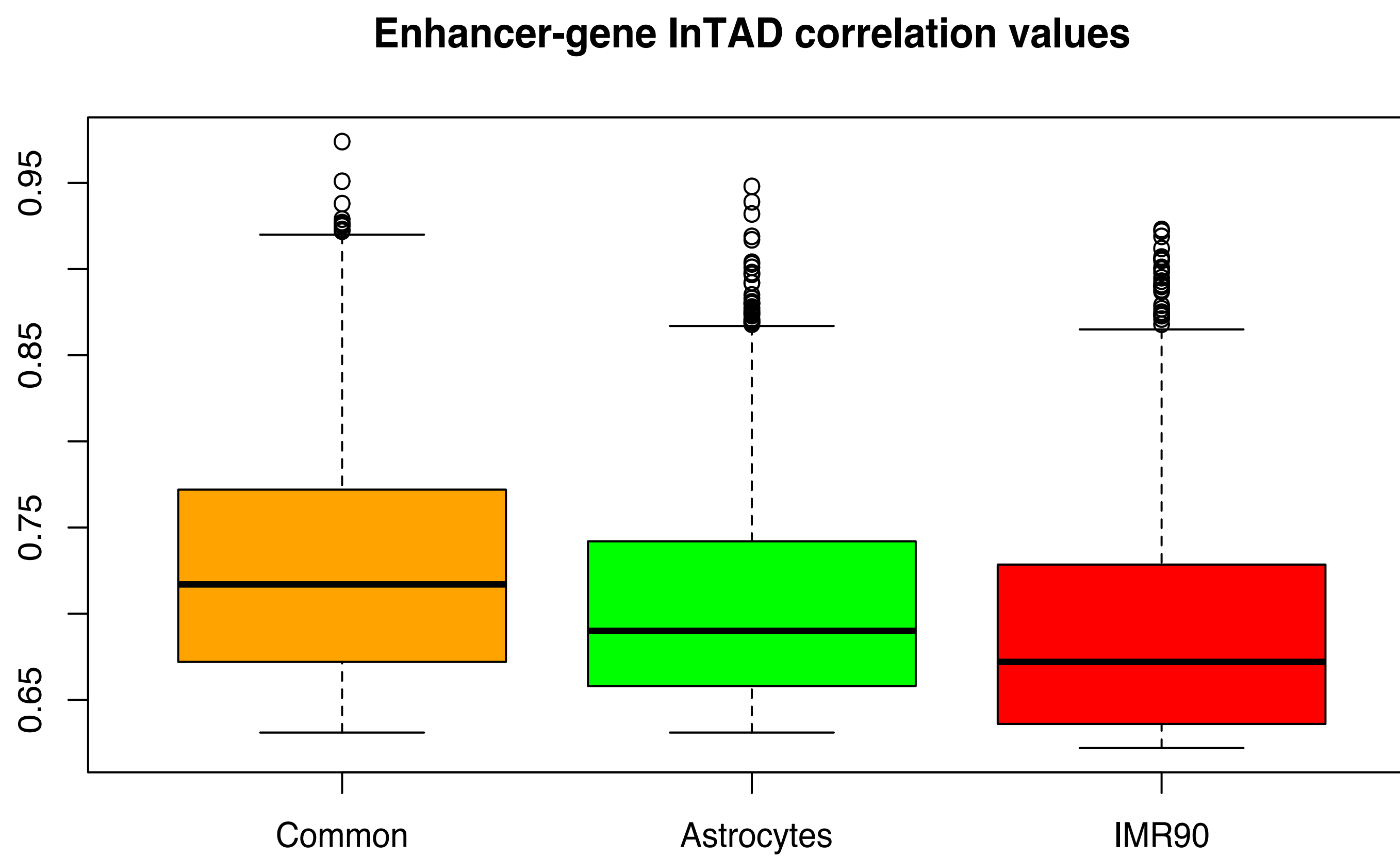

Supplement: Supplementary file 2 — Figure S2. A) The variance between the sizes of TADs derived from IMR90 and cerebellum astrocytes. B) Venn-diagram showing the number and overlap of enhancer associated genes identified in ependymoma tumors using TADs derived from IMR90 or cerebellum astrocytes, respectively. C) Boxplot summarizing the enhancer-gene correlation values obtained when considering TADs common between IMR90 and cerebellum astrocytes or TADs specific to cerebellum astrocytes or IMR90, respectively. (PDF 88 kb) [file 12859_2019_2655_MOESM2_ESM.pdf]
